# In Situ Synthesis of Silver Nanoparticles on Cellulose Fibers Using D-Glucuronic Acid and Its Antibacterial Application

**DOI:** 10.3390/ma12193101

**Published:** 2019-09-23

**Authors:** Guangxue Chen, Linjuan Yan, Xiaofang Wan, Qiankun Zhang, Qing Wang

**Affiliations:** State Key Laboratory of Pulp and Paper Engineering, South China University of Technology, Guangzhou 510640, China; chengx@scut.edu.cn (G.C.);

**Keywords:** green synthesis, silver nanoparticles, D-glucuronic acid, cellulose fibers, antibacterial properties

## Abstract

The development of ecofriendly procedures to avoid the use of toxic chemicals for the synthesis of stable silver nanoparticles (AgNPs) is highly desired. In the present study, we reported an eco-friendly and green technique for in situ fabrication of AgNPs on bleached hardwood pulp fibers (bhpFibers) using D-glucuronic acid as the only reducing agent. Different amounts of D-glucuronic acid were introduced and its effect on the size and distribution of AgNPs on the bhpFibers was discussed. The morphology and structures of bhpFibers@AgNPs were proved by electron microscope-dispersive spectroscopy (SEM-EDS), X-ray diffraction (XRD), Fourier transform infrared (FT-IR) and X-ray photoelectron spectroscopy (XPS). Then, a series of bhpFibers@AgNPs with different AgNPs loadings were also prepared by adjusting the concentration of the AgNO_3_ solution. After a papermaking process via vacuum filtration, the prepared papers displayed an outstanding antibacterial performance against *Escherichia coli* (gram -negative) and *Staphylococcus aureus* (gram-positive). It is foreseeable that the bhpFibers@AgNPs have a promising application in the field of biomedical.

## 1. Introduction

AgNPs have been widely accepted as the most efficacious metal with antimicrobial properties [1,2,3,4,5]. However, the dissociative AgNPs or silver ions may cause inevitable harm to human health and the environment, which greatly hinders the practical application [6]. Therefore, AgNPs have generally been in situ generated onto various polymeric matrices [7,8,9,10]. These silver-based nanocomposites possess excellent antimicrobial properties against a wide scope of pathogens, which play vital roles in medical antibacteria [11,12,13], wound treatment [14,15], food packaging and textiles [16,17]. Since the size, morphology and structure of AgNPs are strongly related to their antimicrobial properties, the synthesis process should be technologically controlled.

Among all the polymer matrices, cellulose has attracted much attention due to the advantages of abundant resources [18,19,20,21] and rich hydroxyls in its molecular structure [22,23]. Recently, methods have been developed for the fabrication of cellulose/AgNP nanocomposites. Zhu et al. [24] presented an in situ synthesis of AgNPs in pristine cellulose fibers using NaBH_4_ as the reduction agent. The mean size and size distribution of AgNPs were adjusted by changing the concentration of NaBH_4_. Maria et al. [25] developed bacterial cellulose/silver nanocomposites based on different reductants of hydrazine and hydroxylamine, with the help of such additional agents as gelatin or polyvinylpyrrolidone. Li et al. [26] fabricated cellulose–silver nanocomposites using ethylene glycol (EG) as a reducing agent with a microwave-assisted method. Clearly, the use of organic solvents or the use of toxic chemicals as reducing agents during the preparation are unfavorable for the environment, which largely limits their actual application in biomedical fields. Thus, non-toxic green reductants such as glucose [27] and ascorbic acid are commonly chosen for the synthesis of AgNPs in matrices. However, due to the weak reduction of these reducing agents, larger particle sizes or clusters of AgNPs in cellulose have been observed, which lead to poor antibacterial effects [28]. For example, Li et al. [29] found that polyhedral silver particles with a size of 250 nm exhibited relatively poor dispersion in the cellulose matrix when using ascorbic acid as a reducing agent. In order to further improve the greenness of the in situ generation of AgNPs, using some biological substances, including plant and plant extracts [30,31], as reducing agents has become a new trend. Aladpoosh et al. [32] prepared AgNPs on cotton fabric using ashes of *S. rosmarinus*. The results showed that aggregation of particles in the matrix was unavoidable. Sri Ramkumar et al. [33] first prepared a seaweed extract that was obtained from seaweed by using a mixer grinder, then AgNPs were synthesized by bio-reduction of AgNO_3_ with the prepared seaweed extract. It is evident that the introduction of these plant reducing agents might cause unexpected aggregation of AgNPs and cumbersome processes. Therefore, it is still a challenge to prepare the AgNPs with small size and uniform distribution in the matrix with green and ecofriendly techniques.

D-glucuronic acid (DLA) might be a most attractive candidate for the synthesis of silver nanoparticles among these chemical reducing agents because of its outstanding features. First, DLA is abundant in many biological systems and is non-toxic [34], which is crucial for ‘green chemistry ’. Besides, DLA has abundant hydroxyl groups and a terminal aldehyde group in its units [35,36], which might be exploited as glucose. Most of all, DLA is water soluble and possesses negatively charged carboxyl groups, which might electrostatically interact with Ag^+^ and promote the distribution of Ag^+^ in the cellulose matrix [36,37]. It also has been reported that DLA was able to reduce Cr^+5^ to Cr^+3^ [38]. From this point, we can except that the DLA can also be used to reduce silver ions to AgNPs on cellulose fibers.

Herein, a one-step green hydrothermal technique for in situ fabrication of AgNPs on bleached hardwood pulp fibers (bhpFibers) was proposed by only using DLA as the reducing agent. Different amounts of DLA were introduced and its effect on the size and distribution of AgNPs on the bhpFibers was discussed. Surface morphology and structures of bhpFibers before and after surface modification were characterized using SEM images, XRD patterns, FT-IR spectra and EDS mapping. Then, a series of bhpFibers@AgNPs with different AgNPs loadings were also prepared by adjusting the concentration of the AgNO_3_ solution. After the papermaking process, the antibacterial properties of bhpFibers@AgNPs against the *Escherichia coli* (gram-negative) and *Staphylococcus aureus* (gram- positive) were separately investigated.

## 2. Experimental

### 2.1. Materials

A pulp board made from poplar was supplied by Yueyang Paper Co., Ltd., Yueyang, China. Silver nitrate (AgNO_3_, AR) was purchased from Sinopharm Chemical Reagent Co, Ltd. (Shanghai, China). The ammonia solution (NH_3_⋅H_2_O, 25 wt%) was provided by Guangzhou Chemical Reagents Factory (Guangzhou, China). Sodium hydroxide (NaOH, > 98 %) and D-glucuronic acid (DLA) were obtained from Sinopharm Chemical Reagent Company (Shanghai, China). All the reagents were analytical purity used without further purification. All the water used was deionized water.

### 2.2. Pretreatment of Bleached Hardwood Pulp 

The bleached hardwood pulp was first defibered (100 rpm/min) using a fluffer to separate the interwoven fibers in water without altering the original structure of the fibers. Then bleached hardwood pulp fibers (bhpFibers) with a beating degree of 48 °SR could be obtained after the beat with a speed of 25,000 rpm by the PFI pulping machine (Mark VI, 621, Hamjern Maskin, Oslo, Norway).

### 2.3. Synthesis of bhpFibers@AgNPs 

**Scheme 1:**
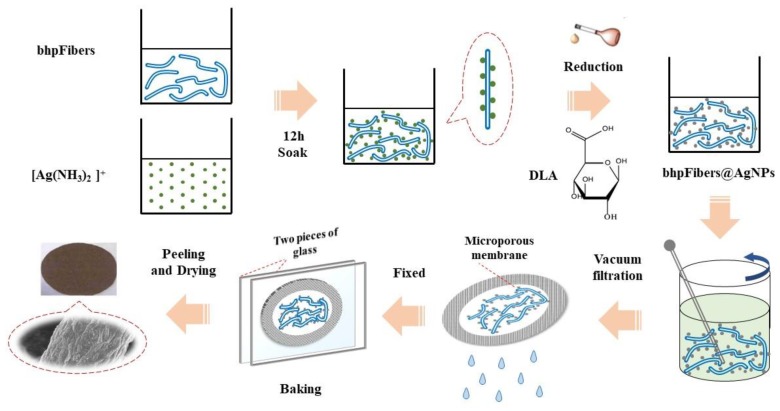
Schematic of the fabrication of bhpFibers@AgNPs and bhpFibers@AgNP-based paper. AgNPs: Stable silver nanoparticles; bhpFibers: Bleached hardwood pulp fibers.

The bhpFibers@AgNPs were prepared through reduction of AgNPs in situ on bhpFibers (Scheme 1). In a typical procedure, 0.2 g of dried bhpFibers were dispersed in 30 mL of deionized water. Then, 10 mL of Ag(NH_3_)_2_^+^ solutions with appropriate concentrations were added into the above suspension and transferred to the refrigerator overnight. Subsequently, 10 mL of different concentrations (0, 0.1, 0.2, 0.4 and 0.6 mg/mL) of DLA solutions were dropwise introduced in 20 minutes and conducted for 2 h at 50 °C. The reaction mixtures were then centrifuged (8000 rpm, 3 min) and rinsed with deionized water three times to obtain bhpFibers@AgNPs composites.

The Ag(NH_3_) solutions were prepared by adding 25 wt% ammonia water dropwise into 20, 40, 60 and 80 mM silver nitrate solutions until the pH of the mixtures was about 11. A few drops of NaOH were added to the obtained Ag(NH_3_)_2_^+^ solutions to adjust the pH value to 11.5. AgNPs on bhpFibers were synthesized according to the following reactions:

(1) $${\mathrm{AgNO}}_{3}+2{\mathrm{NH}}_{3}\cdot H_{2}O⇌\mathrm{Ag}({\mathrm{NH}}_{3}{{)}_{2}}^{+}+{{\mathrm{NO}}_{3}}^{-}+2H_{2}O$$

(2) $$2[\mathrm{Ag}({\mathrm{NH}}_{3}{)}_{2}]\mathrm{OH}+C_{6}H_{10}O_{7}\left(\mathrm{DLA}\right)⇌C_{5}H_{9}O_{6}{\mathrm{COONH}}_{4}+2\mathrm{Ag}↓+3{\mathrm{NH}}_{3}+H_{2}O$$

### 2.4. Fabrication of bhpFibers@AgNP-based paper

As shown in Scheme 1, the bhpFibers@AgNP-based paper was fabricated via a vacuum filtration process. Typically, the prepared bhpFibers@AgNPs was dispersed in 100 mL deionized water to form a stable pulp suspension under gentle stirring. Then, the suspension was poured into the filtration funnel with a Φ = 0.22 μm microporous membrane. After being vacuum-dried at 60 °C for 12 h, the bhpFibers@AgNP-based paper was successfully prepared. A series of papers with different amounts of AgNPs were also prepared by adjusting the concentration of silver ions in the solution. 

### 2.5. Characterization

Morphological characteristics and energy dispersive spectroscopy (EDS) were captured with emission scanning electron microscopy (SEM, Zeiss Merline, Oberkochen, Germany). Crystal structures were performed in the range of 4° to 90° on X-ray diffraction (XRD, Philips PW3040/00 X’Pert MPD, Westborough, MA, USA) using a diffractometer with Cu Kα radiation at the 3 kW. Chemical structures were characterized by FT-IR spectra (FTIR-ATR, Bruker VerTex 70, Billerica, Massachusetts, Germany). Chemical compositions were performed on X-ray photoelectron spectroscopy (XPS, Escalab 250 spectrometer, Thermo Electron Corporation, Waltham, MA, America). The thermal gravimetric analyses were investigated by a TGA Instruments (TGA, TA Q500, New Castle county, Delaware, America) heating from 25 to 700 °C with a heating rate of 10 °C/min in a nitrogen atmosphere at a flow rate of 40 mL min^−1^. The silver content was measured by inductively coupled plasma atomic emission spectrometry (ICP-ms, Hitachi Z-2000, Tokyo, Japan).

### 2.6. Antimicrobial Activity Test

Antibacterial detections of the solid plate medium (inhibition zone measurement) are as follows: 10 mL of the melted solid Luria-Bertani (LB) medium was poured into a Petri dish (90 mm in diameter) before waiting for solidification. Then, *Escherichia coli* (*E. coli*) DH5α or *Staphylococcus aureus* (*S. aureus*) was cultured in a liquid LB medium at 220 rpm and 37 °C until the optical density (OD) 600 was about 0.6, and the concentration of bacteria reached a 108 colony-forming unit (CFU)/mL. The obtained *E. coli* or *S. aureus* suspension was taken out to be spread on LB agar plates three times with sterile tweezers. Finally, the prepared bhpFiber-based paper and bhpFibers@AgNP-based paper were placed on the culture medium for full contact. The inoculated microorganisms were cultured at 37 °C for 24 hours. The antibacterial property of the composites paper was evaluated by the size of the inhibitory zone.

## 3. Results and Discussion

### 3.1. Effect of DLA on in-situ preparation of AgNPs on bhpFibers

**Figure 1:**
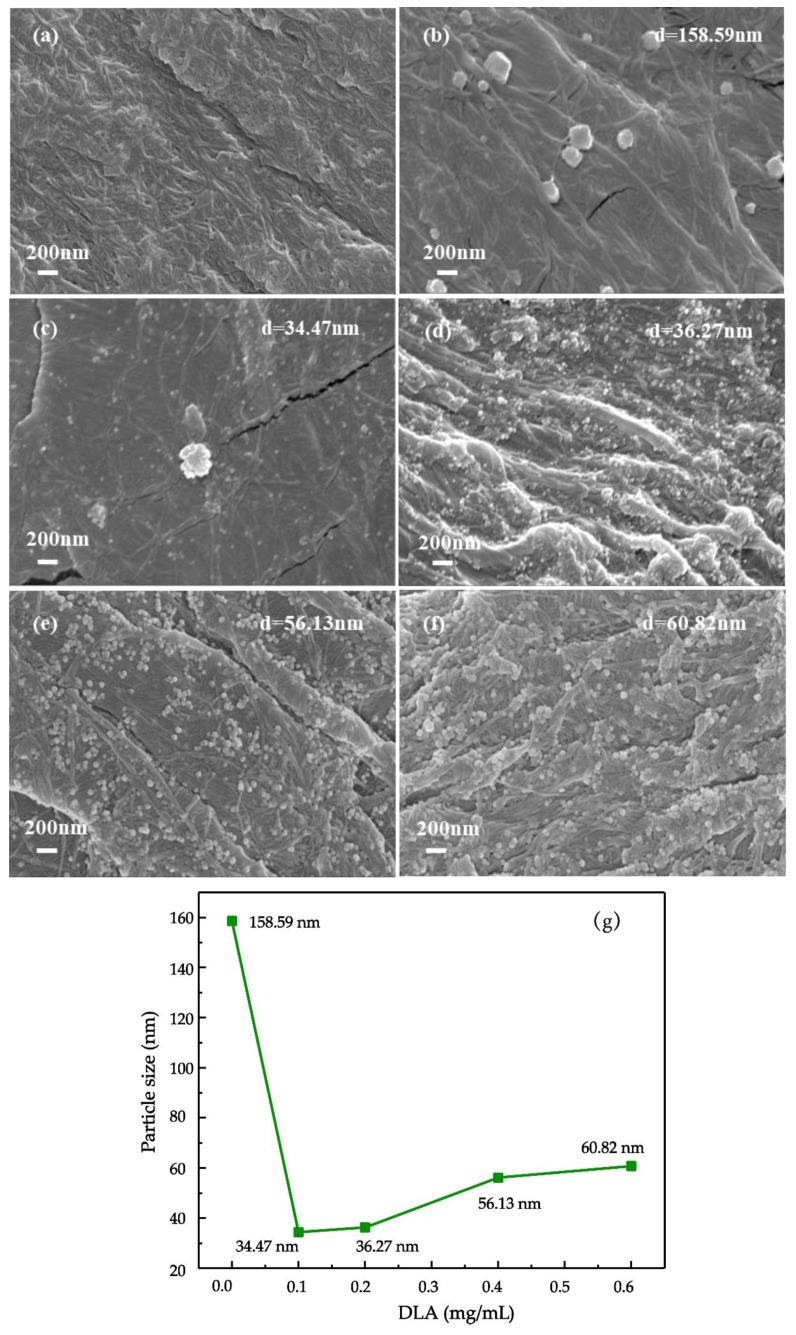
The SEM images of (**a**) bhpFibers and bhpFibers@AgNP-based paper prepared by 20 mM AgNO_3_ and different concentrations of DLA: (**b**) 0 mg/mL, (**c**) 0.1 mg/mL, (**d**) 0.2 mg/mL, (**e**) 0.4 mg/mL and (**f**) 0.6 mg/mL; (**g**) silver particle sizes at different concentrations of DLA.

SEM was used as a powerful tool to vividly illustrate the morphologies of bhpFibers before and after surface modification. The morphological features and particle size distribution of AgNPs that varied with the concentration of DLA are exhibited in Figure 1 and Figure S1; the pristine bhpFibers in Figure 1a are interwoven by a three-dimensional network composed of interpenetrated cellulose fibers. The interconnected structure increased the fibers’ surface roughness, affording good support for the anchoring of AgNPs. After in situ reduction of AgNPs without the aid of DLA, the bhpFiber surfaces were distributed with obviously large particles or clusters of AgNPs of 158.59 nm, as shown in Figure 1b and Figure S1b. When the concentration of DLA was 0.1 mg/mL, the AgNPs with a smaller size of average diameter 34.47 nm gradually appeared in Figure 1c and Figure S1c, in which most of the particles were spheres, but there still existed some large aggregated silver particles. When the DLA amount increased to 0.2 mg/mL, the obtained nanoparticles of bhpFibers had a uniform size distribution and an average diameter of 36.27 nm, as shown in Figure 1d and Figure S1d; they were all spheres and well separated from each other owing to the sufficient DLA molecules, suggesting that the addition of DLA was beneficial for the reduction of aggregated particles and the formation of nanoparticles with uniform and small particle size. With the amount of DLA increasing to 0.4 and 0.6 mg/mL, silver nanoparticles with a larger size, of average diameters 56.13 nm and 60.82 nm, were prepared on bhpFibers as shown in Figure 1e,f and Figure S1e,f. This might be due to more reducing agents causing faster reduction of Ag(NH_3_)_2_^+^ and then forming large AgNPs [27]. Figure 1g shows intuitively the particle size of AgNPs on bhpFibers under different amounts of DLA, indicating that the appropriate concentration of DLA can be exploited to control the different sizes of AgNPs. Meanwhile, the smaller AgNPs with larger specific surface areas are accessible for the interaction between AgNPs and bacteria, affording higher antimicrobial effects than larger AgNPs [28]. Based on the results of Figure 1, 0.2 mg/mL of DLA was conducive to the generation of AgNPs with the smallest particle size and uniform distribution on bhpFibers. Compared to the silver particles (80–120 nm) reduced by glucose [27,39], DLA acts as a reducing agent to achieve smaller particle size (36.27 nm) and better dispersion of silver nanoparticles in the cellulose matrix. This may be because DLA molecules could bind to Ag ions through carboxyl group chelation, which facilitates the reduction of Ag ions [40]. 

### 3.2. Characterization of the Pristine bhpFibers and bhpFibers@AgNPs Composites

**Figure 2:**
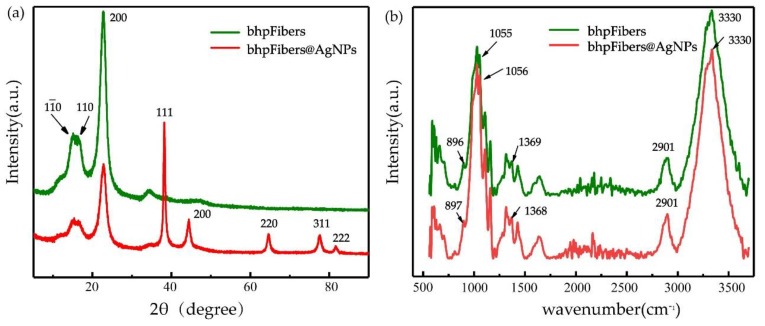
XRD patterns (**a**) and FT-IR spectra (**b**) of bhpFibers and bhpFibers@AgNP-based paper.

The crystalline phases of pristine bhpFibers and bhpFibers@AgNP-based paper were characterized by X-ray diffractometry (XRD), shown in Figure 2a. The XRD results show that the bhpFibers had three diffraction characteristic peaks at 2θ = 14.8, 16.5 and 22.5 corresponding to the typical (110), (110) and (200) planes of cellulose fibers (JCPDS50-0926), respectively [41]. Compared with bhpFibers, the bhpFibers@AgNPs showed typical XRD pattern of Ag nanocrystals on different crystal planes. Moreover, there were five peaks at 2θ = 38.1°, 44.3°, 64.4°, 77.5° and 81.6° in the patterns. These peaks were in accordance with the JCPDS database (No. 89-3722), which corresponded to the (111), (200), (220), (311) and (222) lattice planes of Ag [42], suggesting the synthesis of silver in the crystalline state by using DLA as the reducing agent was successful. In addition, no typical peaks of substance such as Ag_2_O were observed, demonstrating that the pure AgNPs were synthesized on the surface of bhpFibers. 

The structures of the pristine bhpFibers and bhpFibers@AgNP-based paper were investigated by FT-IR in Figure 2b. As for the pristine bhpFibers, the peak at 3330 cm^−1^ was attributed to the characteristic absorption of O–H stretching. The vibration bands at 2901 cm^−1^ and 1368 cm^−1^ can be assigned to the asymmetrical and symmetrical stretching of the C-H group, respectively. The absorption band at 1055 cm^−1^ corresponded to C–O–C stretching mode from the glucosidic units [26]. The peak at 894 cm^−1^ was related to the C–H rocking vibration of cellulose. These bands appeared in both the pristine bhpFibers and the modified cellulose fibers, suggesting the chemical structure of the cellulose was barely changed. No new peaks appeared in the bhpFibers@AgNPs, indicating that the interaction between cellulose fibers and AgNPs was a physical adsorption.

**Figure 3:**
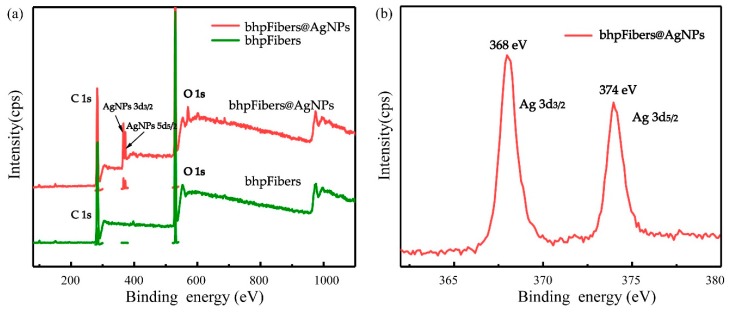
(**a**) XPS spectra of bhpFibers and bhpFibers@AgNP-based paper; (**b**) Ag3d XPS spectrum of bhpFibers@AgNP-based paper.

The XPS technique of pristine bhpFibers and bhpFibers@AgNP-based paper could provide further information about the structure and chemical state of AgNPs on cellulose fibers. As depicted in Figure 3a, the pristine bhpFibers showed the binding energy spectra of C1 s peaks (288 eV) and O1 s peaks (533 eV), respectively, which can be inferred to lignocellulosic characteristic peaks of cellulose materials [30]. After surface modification, Ag3d peaks were observed in the bhpFibers@AgNPs, demonstrating the formation of AgNPs. A high resolution XPS spectra of Ag3d (Figure 3b) was undertaken to analyze the valence state of Ag on the surface of cellulose fibers, it can be seen that the peaks of Ag3d_3/2_ at 368 eV and Ag3d_5/2_ at 374 eV appeared, demonstrating that the stable zero-valence AgNPs had been successfully loaded on the bhpFibers surface [43,44].

**Figure 4:**
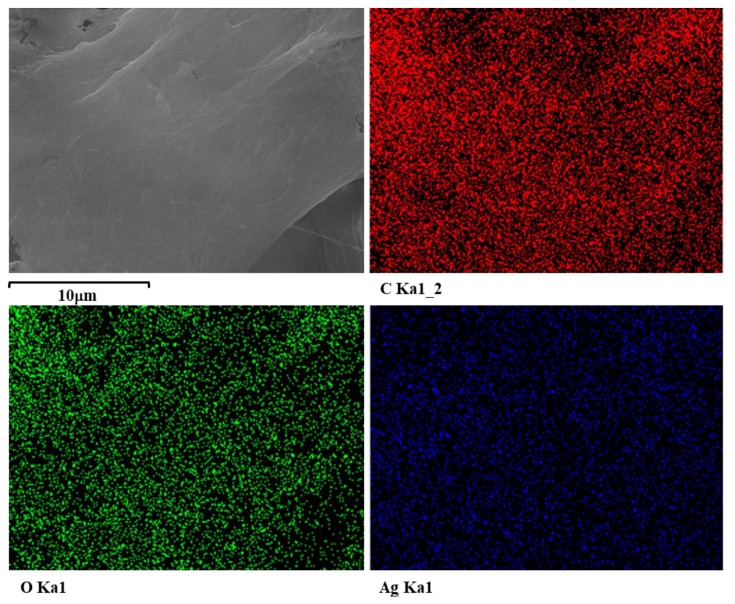
EDS elemental mapping images of the bhpFibers@AgNP-based paper.

Besides, X-ray energy dispersive spectroscopy mapping (EDS), described in Figure 4, shows that the elements of C, O and Ag were uniformly distributed on the surface of fibers, which also proved that bhpFibers were successfully coated with AgNPs.

### 3.3. The effect of AgNO_3_ on the deposition amounts of AgNPs on bhpFibers

**Figure 5:**
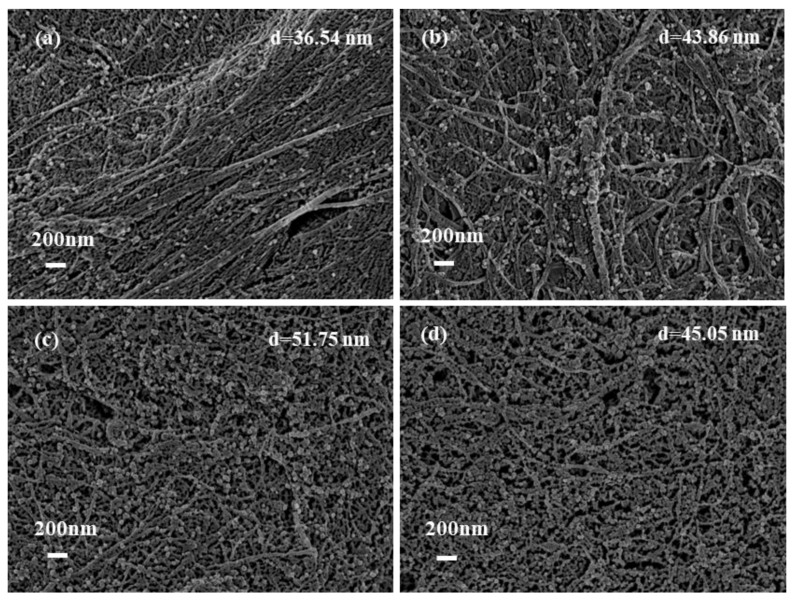
The SEM images (a–d) of bhpFibers@AgNP-based paper prepared by 0.2 mg/mL DLA and different concentrations of AgNO_3_: (**a**) 20 mM, (**b**) 40 mM, (**c**) 60 mM, (**d**) 80 mM.

Subsequently, the effects of the concentration of AgNO_3_ were explored by mixing different concentrations of AgNO_3_ (20–80 mM) with 0.2 mg/mL of DLA. As shown in Figure 5, when the concentration of AgNO_3_ increased from 20 to 80 mM, the size of the prepared AgNPs on bhpFibers increased slightly from 36.54, 43.86 and 51.75 to 45.05 nm. The AgNPs content in the composites could be determined by the amount of residual at 700 °C in TGA curves (Figure S2) and the weight percentages of Ag were calculated to be 9.36%, 10.26%, 12.91% and 11.72%, demonstrating that more silver nanoparticles were incorporated into bhpFibers. This result supported the results of the scanning electron microscopy in Figure 5. Moreover, the colour of the obtained papers and ICP-ms analysis of the AgNPs content on bhpFibers was also measured, as shown in Table 1. It can be seen that the paper colour darkened with the AgNPs content when the concentration of AgNO_3_ was within 60 mM. When more AgNO_3_ (higher than 60 mM) was added, the impregated AgNPs on the bhpFibers decreased a little. A similar phenomenon was reported for the cellulose/AgNPs membrane [27]. It is noteworthy that all the AgNPs on bhpFibers in Figure 5 were highly dispersed and no visible agglomeration occurred, which was attributed to the strong interaction between hydroxyl and oxygen-containing groups on the cellulose with the AgNPs [40]. According to the above analysis, 60 mM of AgNO_3_ was sufficient for achieving the desired silver content on bhpFibers surfaces. 

### 3.4. Antibacterial Properties of bhpFibers@AgNP-Based Paper

**Figure 6:**
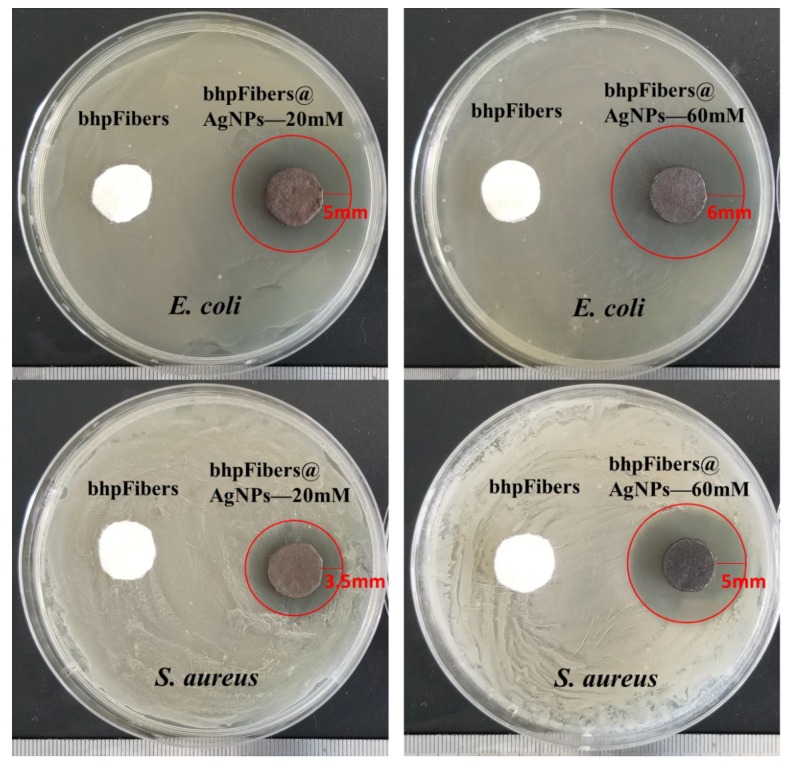
The antibacterial property of bhpFibers and bhpFibers@AgNP-based paper generated using aq. AgNO_3_ solutions of concentrations 20 and 60 mM, respectively, against *E. coli and S. aureus*.

It has been reported that the antibacterial properties of the silver-based nanocomposites are related to the mass of AgNPs in the matrix [24,26]. Herein, the antibacterial activities of bhpFibers@AgNP-based paper prepared by 20 mM and 60 mM AgNO_3_ were tested on Gram- negative *E. coli* and Gram-positive *S. aureus*, respectively. As presented in Figure 6, no inhibition zones were observed nearby the pristine fiber-based papers, indicating that the papers did not show any antibacterial activity. The inhibition zones of the bhpFibers@AgNP-based papers with 20 mM AgNO_3_ for *E. coli* and *S. aureus* were 5 mm and 3.5 mm, respectively, while the inhibition zones of the bhpFibers@AgNP-based paper with 60 mM silver nitrate for *E. coli* and *S. aureus* were 6 mm and 5 mm. These results demonstrated that the prepared papers showed outstanding antibacterial activity against both *E. coli* (Gram-negative) and *S. aureus* (Gram-positive). This is because AgNPs can penetrate the cytoplasm of *E. coli* and *S. aureus*, and interact with cell components, causing damage to bacteria. Moreover, with the increasing of AgNPs in papers, the inhibition zones against *E. coli* and *S. aureus* increased simultaneously. These results clearly indicated that the antibacterial ability only stems from the AgNPs impregnating inside bhpFibers. A better antibacterial effect was obtained accompanied by the increase of silver content in the bhpFibers.

## 4. Conclusions

In summary, an environmentally benign synthesis method was provided for in situ fabrication of AgNPs on bhpFibers using DLA as the only reducing agent, which was then used for the preparation of antibacterial papers. SEM results indicated that the AgNPs were homogeneously dispersed in the matrix and AgNPs were efficiently synthesized by means of XRD and XPS measurement. FT-IR indicates that the interaction between cellulose fibers and AgNPs was a physical adherence. Different amounts of DLA were introduced and its effect on the size and distribution of AgNPs on the bhpFibers was discussed. It was found that 0.2 mg/mL DLA was most conducive to the generation of AgNPs with the smallest particle size (36.27 nm) and uniform distribution. Based on this, a serial of bhpFibers@AgNPs with different amounts of AgNPs were prepared by adjusting the concentration of AgNO_3_. The results indicate that 60 mM of AgNO_3_ was sufficient for achieving the desired silver content on bhpFibers surfaces. Moreover, the bhpFibers@AgNP-based paper exhibited outstanding antimicrobial performance against *E. coli* (gram negative) and *S. aureus* (gram positive). Therefore, it is believed that this eco-friendly and green synthesis approach for fabrication of the bhpFibers@AgNPs has a promising application in the field of antibacterials.

## Figures and Tables

**Table 1:** The color and the AgNPs content on the samples.

Sample	bhpFibers	bhpFibers@AgNPs-20 mM	bhpFibers@AgNPs-40 mM	bhpFibers@AgNPs-60 mM	bhpFibers@AgNPs-80 mM
AgNPs content	0	8.2 mg/g	22.4 mg/g	33 mg/g	31.8 mg/g
Color	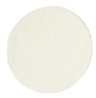	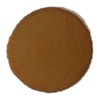	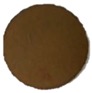	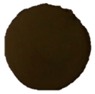	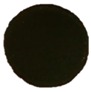

## Supplementary Materials

The following are available online at https://www.mdpi.com/1996-1944/12/19/3101/s1, Figure S1: Size distribution of bhpFibers@AgNPs prepared by 20 mM AgNO_3_ and different concentrations of DLA: (b) 0 mg/mL, (c) 0.1 mg/mL, (d) 0.2 mg/mL, (e) 0.4 mg/mL and (f) 0.6 mg/mL, Figure S2: TG curves of pristine bhpFibers and bhpFibers@AgNPs prepared by 0.2 mg/mL DLA and different concentrations of AgNO_3_: 20 mM, 40 mM, 60 mM and 80 mM.
