# Supplementary material for: In Situ Synthesis of Silver Nanoparticles on Cellulose Fibers Using D-Glucuronic Acid and Its Antibacterial Application
Source: Materials (Basel). 2019 Sep 23;12(19):3101. doi: 10.3390/ma12193101 (PMC6803989; doi:10.3390/ma12193101)
Supplement: Supplementary file 1 [file materials-12-03101-s001.pdf]

materials

MDPI

Article

In Situ Synthesis of Silver Nanoparticles on Cellulose Fibers Using D-Glucuronic Acid and Its Antibacterial Application

Guangxue Chen, Linjuan Yan, Xiaofang Wan *, Qiankun Zhang and Qing Wang *

State Key Laboratory of Pulp and Paper Engineering, South China University of Technology, Guangzhou 510640, China; chengx@scut.edu.cn (G.C.); felinjuan1025@mail.scut.edu.cn (L.Y.); 201820125931@mail.scut.edu.cn (Q.Z.)

* Correspondence: wangqing@szyuto.com (Q.W.); wanxf@scut.edu.cn (X.W.)

Received: 25 August 2019; Accepted: 19 September 2019; Published: date

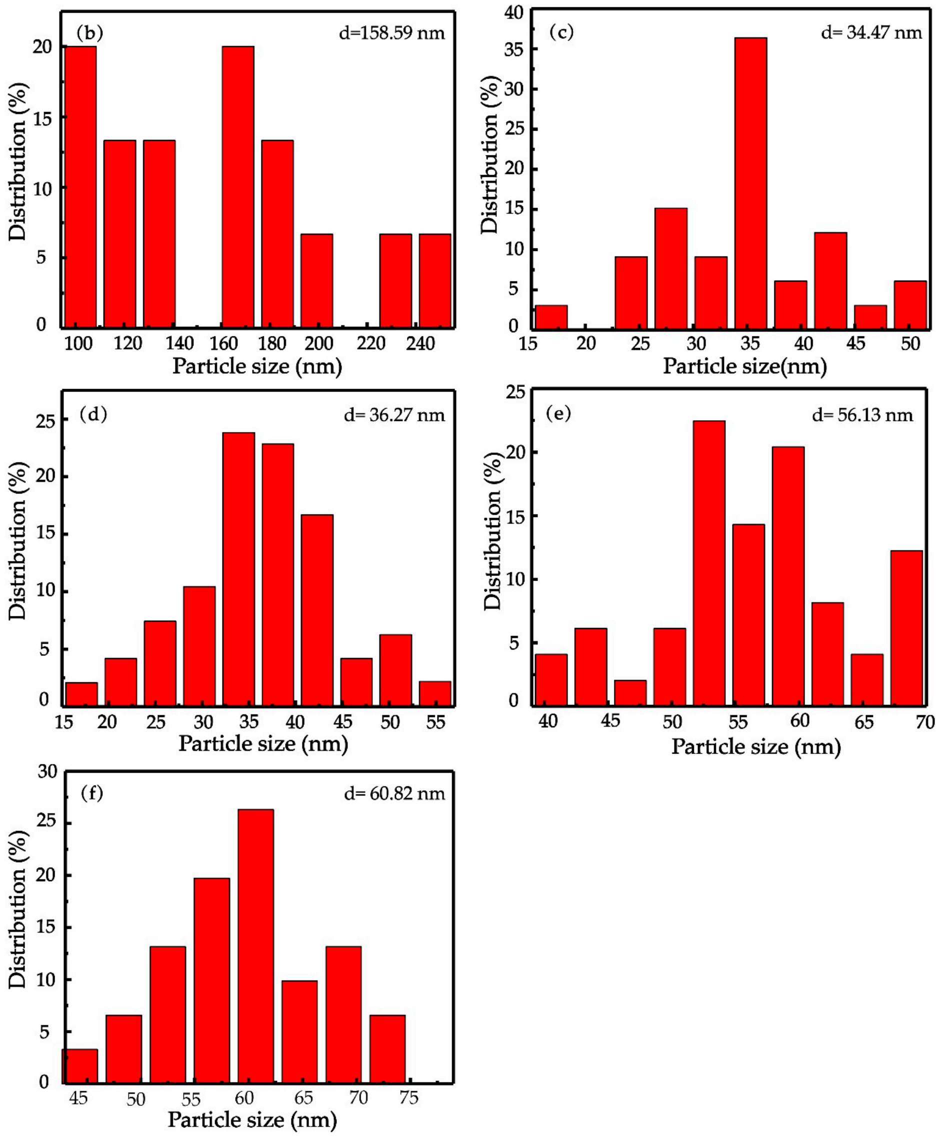

Figure S1. Size distribution of bhpFibers@AgNPs prepared by 20 mM $AgNO_3$ and different concentrations of DLA: (**b**) 0 mg/mL, (**c**) 0.1 mg/mL, (**d**) 0.2 mg/mL, (**e**) 0.4 mg/mL and (**f**) 0.6 mg/mL.

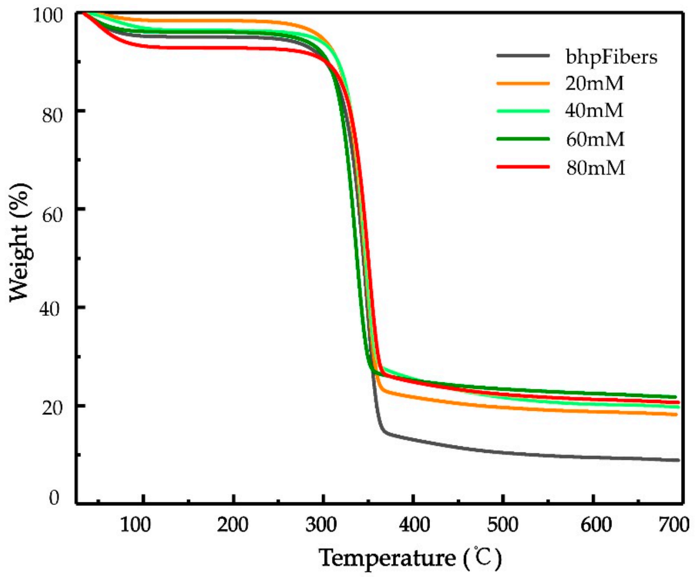

Figure S2. TG curves of pristine bhpFibers and bhpFibers@AgNP-based paper prepared by 0.2 mg/mL DLA and different concentrations of $AgNO_3$: 20 mM, 40 mM, 60 mM and 80 mM.
